# *N*-ethyl-*N*-Nitrosourea (ENU) Induced Mutations within the *Klotho* Gene Lead to Ectopic Calcification and Reduced Lifespan in Mouse Models

**DOI:** 10.1371/journal.pone.0122650

**Published:** 2015-04-10

**Authors:** Christopher T. Esapa, Fadil M. Hannan, Valerie N. Babinsky, Paul Potter, Gethin P. Thomas, Peter I. Croucher, Matthew A. Brown, Steve D. M. Brown, Roger D. Cox, Rajesh V. Thakker

**Affiliations:** 1 Academic Endocrine Unit, Radcliffe Department of Medicine, Oxford Centre for Diabetes, Endocrinology and Metabolism (OCDEM), University of Oxford, Oxford, United Kingdom; 2 Medical Research Council (MRC) Mammalian Genetics Unit and Mary Lyon Centre, MRC Harwell, Harwell Science and Innovation Campus, Harwell, United Kingdom; 3 Department of Musculoskeletal Biology, Institute of Ageing and Chronic Disease, University of Liverpool, Liverpool, United Kingdom; 4 University of Queensland Diamantina Institute, Translational Research Institute, Princess Alexandra Hospital, University of Queensland, Brisbane, Australia; 5 Garvan Institute for Medical Research, Sydney, Australia; The University of Tokyo, JAPAN

## Abstract

Ectopic calcification (EC), which is the pathological deposition of calcium and phosphate in extra-skeletal tissues, may be associated with hypercalcaemic and hyperphosphataemic disorders, or it may occur in the absence of metabolic abnormalities. In addition, EC may be inherited as part of several monogenic disorders and studies of these have provided valuable insights into the metabolic pathways regulating mineral metabolism. For example, studies of tumoural calcinosis, a disorder characterised by hyperphosphataemia and progressive EC, have revealed mutations of fibroblast growth factor 23 (*FGF23*), polypeptide N-acetyl galactosaminyltransferase 3 (*GALNT3*) and klotho (*KL*), which are all part of a phosphate-regulating pathway. However, such studies in humans are limited by the lack of available large families with EC, and to facilitate such studies we assessed the progeny of mice treated with the chemical mutagen *N*-ethyl-*N*-nitrosourea (ENU) for EC. This identified two mutants with autosomal recessive forms of EC, and reduced lifespan, designated *Ecalc1* and *Ecalc2*. Genetic mapping localized the *Ecalc1* and *Ecalc2* loci to a 11.0 Mb region on chromosome 5 that contained the klotho gene (*Kl*), and DNA sequence analysis identified nonsense (Gln203Stop) and missense (Ile604Asn) *Kl* mutations in *Ecalc1* and *Ecalc2* mice, respectively. The Gln203Stop mutation, located in KL1 domain, was severely hypomorphic and led to a 17-fold reduction of renal *Kl* expression. The Ile604Asn mutation, located in KL2 domain, was predicted to impair klotho protein stability and *in vitro* expression studies in COS-7 cells revealed endoplasmic reticulum retention of the Ile604Asn mutant. Further phenotype studies undertaken in *Ecalc1* (*kl^203X/203X^*) mice demonstrated elevations in plasma concentrations of phosphate, FGF23 and 1,25-dihydroxyvitamin D. Thus, two allelic variants of *Kl* that develop EC and represent mouse models for tumoural calcinosis have been established.

## Introduction

Ectopic calcification (EC) is characterized by the pathological deposition of calcium and phosphate in extra-skeletal tissues, and represents a major cause of adverse cardiovascular outcomes and mortality [1]. Two types of EC, referred to as metastatic and dystrophic EC, are recognised. Metastatic EC is associated with metabolic abnormalities and arises from sustained elevations in circulating calcium and/or phosphate concentrations, which lead to widespread mineral deposition that particularly affects arterial vessels, kidneys, articular cartilage and peri-articular soft tissues [2], and occurs frequently in major chronic diseases such as chronic renal failure [3]. Dystrophic EC occurs in the absence of systemic metabolic derangements and may represent a response to tissue injury, as highlighted by connective tissue disorders such as scleroderma [2]. In addition, EC may be inherited as part of a monogenic disorder and studies of these diseases have provided valuable insights into the molecular basis and metabolic pathways causing EC. For example, studies have highlighted the central role of pyrophosphate as a mineralization regulator, as germline mutations of the ectonucleotide pyrophosphatase/phosphodiesterase 1 (*ENPP1*) gene, which encodes an enzyme mediating the generation of extracellular pyrophosphate, lead to idiopathic infantile arterial calcification [4], whilst germline mutations of the *ANKH* gene, which encodes a transmembrane protein involved in pyrophosphate transport, may lead to chondrocalcinosis [5]. In addition, studies of tumoural calcinosis (TC), an autosomal recessive disorder characterized by the progressive deposition of calcium phosphate crystals in peri-articular and other soft tissues [6], have revealed hyperphosphataemia to be a major promoter of ectopic calcification and delineated a hormonal mechanism regulating circulating phosphate concentrations [6, 7]. Molecular genetic studies of patients and families with TC have identified the occurrence of mutations of either the fibroblast growth factor 23 (*FGF23*), polypeptide N-acetyl galactosaminyltransferase 3 (*GALNT3*), or klotho (*KL*) genes, which all encode proteins involved in a phosphate-regulating pathway [8–13]. Thus, FGF23 is a secreted osteocyte protein, whose post-translational processing requires GALNT3 mediated mucin type O-glycosylation, and which acts on the renal tubule via a membrane protein complex comprising the FGF receptor (FGFR) and a co-receptor, known as klotho, to promote renal phosphate excretion by downregulating type II sodium-phosphate co-transporters. FGF23 also decreases intestinal phosphate absorption by inhibiting the renal vitamin D-1α-hydroxylase (*Cyp27b1*) mediated synthesis of 1,25-dihydroxyvitamin D [6, 7]. The human *KL* gene encodes a parathyroid and renally expressed 1012 amino acid type 1 transmembrane protein with a 980 amino acid extracellular domain comprised of two internal repeat regions, termed KL1 and KL2 [11, 14] that share homology to the β-glycosidase enzyme family [15, 16] and mediate protein-protein interactions with FGFR [17]. Studies aimed at identifying further genetic abnormalities causing EC in humans are hampered by the lack of available large families with monogenic forms of EC that could facilitate positional cloning studies. To overcome these difficulties and facilitate the identification of genetic abnormalities causing EC, we embarked on establishing mouse models using *N*-ethyl-*N*-nitrosourea (ENU), which is a chemical mutagen that causes point mutations by alkylation of nucleic acids leading to mispairing and subsequent single base substitutions during DNA replication [18]. ENU mouse mutants, which can be associated with loss-of-function, hypomorphic, hypermorphic or dominant-negative allelic variants, have been successfully derived for metabolic disorders [19–21]. An example is our previously reported ENU mouse model for TC due to a Trp589Arg missense mutation of the *Galnt3* gene [20]. We now report the identification of two new ENU-induced mouse mutant models for TC, designated *kl* ^*203X*^ and *kl* ^*604N*^ due to mutations located within the *Kl* coding-region. Previously, transgenic mice with hypomorphic *Kl* alleles (*kl/kl* mice) and kidney-specific *Kl* null (*Six-2-kl* ^*-/-*^) mice, that had hyperphosphataemia, EC and shortened lifespan have been reported [11, 22] (Table 1), and thus our findings of these ENU-induced mouse mutants represents the first report of EC mouse models with *Kl* coding sequence mutations, which will help to further elucidate the molecular basis of klotho function and characterise the role of the FGF23-klotho pathway in the renal regulation of phosphate metabolism.

**Table 1:** Comparison of mouse models and patient harbouring klotho mutations.

	*kl* ^*203X/203X*^ **mice**	***kl/kl* mice**	***Six2-kl*** ^*-/-*^ **mice**	**Patient with *KL* mutation**
**Reference**	-	[9, 27, 29]	[28]	[7]
**Mutation type**	Germline (homozygous) nonsense	Germline hypomorphic/null	Kidney-specific null	Germline (homozygous) missense
**Ectopic calcification**	+	+	+	+
**Osteopaenia**	+	+	+	+
**Growth retardation**	+	+	+	-
**Pulmonary emphysema**	+	+	+	-
**Skin atrophy**	+	+	+	-
**Hearing loss**	+	+	NK	-
**Plasma/serum biochemistry:**				
** Calcium**	Normal	↑	↑	↑
** Phosphate**	↑	↑	↑	↑
** Alkaline phosphatase activity**	↑	NK	NK	Normal
**1,25-dihydroxyvitamin D**	↑	↑	↑	↑
** Fibroblast growth factor 23**	↑	↑	↑	↑
** Parathyroid hormone**	NK	↓	↓	↑
** Glucose**	↓	↓	NK	NK

+, present;-, absent; ↓, reduced; ↑, increased; NK, not known.

## Materials and Methods

### Ethics Statement

All animal studies were carried out using guidelines issued by the Medical Research Council in 'Responsibility in the Use of Animals for Medical Research' (July 1993) and UK Home Office Project License Number 30/2433. Experiments were approved by the Medical Research Council Harwell ethics committee.

### Generation and Initial Characterisation of Mutant Mice

Male C57BL/6J mice were treated with ENU and mated with untreated C3H/HeH female mice [18]. The male progeny (G1) were subsequently mated with wild-type C3H/HeH females to generate G2 progeny. The female G2 progeny were then backcrossed to their G1 fathers and the resulting G3 progeny [18] were screened from 2 weeks of age for recessive phenotypes. Mice were fed an expanded rat and mouse no. 3 breeding diet (Special Diets Services, Witham, UK) containing 1.15% calcium, 0.82% phosphate and 4088.65 units/kg vitamin D, and given water ad libitum. Wild-type littermates were used as controls, as these would have similar random assortments of segregating C57BL/6J and C3H alleles, to those of the mutant mice, thereby minimising any strain-specific influences. Initial assessment of the G3 progeny comprised a dysmorphology screen to assess for gross anatomical changes and auditory function [23, 24], followed by digital radiography and the dissection of tissues for histological analysis [20, 25]. Genome-wide mapping was performed once the progeny had been investigated using these procedures.

### Plasma Biochemistry

Blood samples were collected from the retro-orbital sinus after terminal anaesthesia, and plasma was separated by centrifugation at 3000 g for 5 min at 4°C. Plasma samples were analysed for total calcium, inorganic phosphate, urea, albumin, glucose, alkaline phosphatase and alanine transaminase activities, on a Beckman Coulter AU680 semi-automated clinical chemistry analyzer, as described [25]. Plasma calcium was corrected for albumin using the formula: ((albumin-mean albumin) x0.02) + calcium), as reported [25]. Intact FGF23 was quantified using a two-site ELISA kit (Kainos Laboratoties, Tokyo, Japan), and 1,25-dihydroxyvitamin D was measured by a two-step process involving purification by immunoextraction and quantification by enzyme immunoassay (Immunodiagnostic Systems, Boldon, UK), as described [25].

### Imaging by Radiography and Dual-energy X-ray Absorptiometry

Mice or dissected specimens were subjected to digital radiography at 26kV for 3 seconds using a Faxitron MX-20 digital X-ray system (Faxitron X-ray Corporation), and images were processed using the DicomWorks software (http://www.dicomworks.com/), as previously reported [20]. Dissected femora were scanned by dual-energy X-ray absorptiometry (DXA) using a Lunar PIXImus densitometer (GE Medical Systems) and images were processed using the PIXImus software [20].

### Analysis of Auditory Function

Mice were exposed to a high frequency tone stimulus emitted from a handheld click-box, as described [23], and their hearing ability assessed by the presence of a ‘Preyer’ reflex in the pinnae, which flick backwards to indicate normal hearing.

### Histology

Dissected specimens were fixed in 10% formalin, bones decalcified in formical-4 (Decal Chemical Corporation) for 3 days before embedding in paraffin wax. Soft tissue sections (3–4 μm) were stained with haematoxylin and eosin (H&E) or von Kossa, whereas bone sections were stained with van Gieson and alcian blue 8GX, as described [20]. Digital images were obtained using the Nanozoomer 2.0 Digital Pathology system (Hamamatsu Photonics, Welwyn Garden City, UK). Auditory ossicles were dissected from wild-type and mutant mice and their morphology assessed by microscopy.

### Mapping, DNA Sequence Analysis and Genotyping

Genomic DNA was extracted from auricular or tail biopsies, as described [20]. For genome-wide mapping, genomic DNA was amplified by PCR using a panel of 91 single nucleotide polymorphism (SNP) loci arranged in chromosome sets, and the products were analysed by pyrosequencing, as described [20]. Individual exons of *Kl* were amplified from genomic DNA by PCR using gene-specific primers and Taq PCR Mastermix (Qiagen, Crawley, UK), and the PCR products sequenced using BigDye terminator reagents and ABI 3100 sequencer (Life Technologies, Carlsbad, USA). For genotyping, DNA was amplified using Taq PCR Mastermix (Qiagen, Crawley, UK), as described [20]. Primers utilized to amplify exon 1, which contained the *kl* ^*203X*^ mutation were: forward 5’- CCCACTACCGCTTCTCCATA -3’ and reverse 5’- AGTAGGTTGTGGGCAACCAG-3’. Primers utilized to amplify exon 4, which contained the *kl* ^*604N*^ mutation were: forward 5’-GCTAACAGTTGCTCTGTTCTTTG-3’ and reverse 5’- CCACCACTGGAGTGATGTTG -3’. PCR products were digested with *PstI* and *DpnII* restriction enzymes, respectively, and separated by agarose gel electrophoresis before image acquisition using a Gel Doc UV transilluminator (Bio-Rad, Hemel Hempstead, UK), as described [26].

### *In vitro* Studies of Cellular Localisation

Total RNA was isolated from kidneys of wild-type mice using the RNeasy mini kit (Qiagen, Crawley, UK) and 2 μg was used to synthesize cDNA using AffinityScript multiple temperature reverse transcriptase (Agilent Technologies, Edinburgh, UK) using methods previously described [20]. The full-length membrane-bound form of mouse wild-type *Kl* cDNA was amplified with Easy A (Agilent Technologies, Stockport, UK) using the forward primer (5’- CTC AAG CTT GCT CCC GCA GCA TGC TAG CC -3’) and the reverse primer (5’- GCAG AAT TCG CTT ATA ACT TCT CTG GCC TTT C -3’), and the PCR product sub-cloned into pEGFP-N1 (Clontech, Saint-Germain-en-Laye, France) [27]. The *kl* ^*604N*^ mutation was introduced using site-directed mutagenesis with the forward primer 5’- AC TGG GCC CTG AAC TTG CCT CTG GGT -3’ and its reverse complement, and DNA sequence analysis of the constructs was undertaken, using previously reported methods [27]. The wild-type and mutant *Kl* constructs (1μg of each construct) were transiently transfected into COS-7 cells using jetPEI reagent (Polyplus Transfection, Illkirch, France) and expression visualized by immunofluorescence staining using anti-Golgi matrix protein (GM130) (BD Bioscience, Oxford, UK) or mouse anti-protein disulphide isomerase (PDI) (Enzo Life Science, Exeter, UK) under confocal microscopy, as reported [20]. Western blot analysis was performed using equal amounts of proteins from untreated or enzymatically deglycosylated lysates from transfected COS-7 cells [20]. Briefly, lysates were boiled for 10 min in denaturing buffer (0.5% SDS, 40mM DTT), and treated with endoglycosidase (Endo) H (New England Biolabs, Ipswich, UK). Samples were separated by sodium dodecyl sulfate-polyacrylamide gel electrophoresis (SDS-PAGE), electroblotted onto nitrocellulose membrane (GE Healthcare, Little Chalfont, UK), probed with mouse anti-GFP antibody (Roche Diagnostics, Burgess Hill, UK) followed by HRP-conjugated goat anti-mouse IgG (Bio-Rad, Hemel Hempstead, UK) and visualized by electrochemiluminesence (ECL) detection (GE Healthcare, Little Chalfont, UK), as described [28]. The membrane was stripped and re-probed with mouse 12G10 anti-alpha-tubulin antibody (Developmental Studies Hybridoma Bank, University of Iowa) as a loading control.

### *Ex vivo* Gene Expression Studies

Total RNA was isolated from kidneys using the RNeasy mini kit (Qiagen, Crawley, UK) and 2 μg was used to synthesize cDNA using AffinityScript multiple temperature reverse transcriptase (Agilent Technologies, Edinburgh, UK), as described [20]. cDNA templates were amplified by quantitative PCR using SYBR Green (Applied Biosystems, California, USA) and Applied Biosystems 7500 Fast Real-Time PCR System [20], and gene-specific primers for *Kl* (forward: GCTCAACTCTCCCAGTCAGG and reverse: GTGTTCCAGAACCCAGGAAG), *Cyp27b1* (forward: TGGAGTGGACACGGTATCCA and reverse: GGTCCCAGCTGTGATCTCAGA), and the endogenous house-keeping gene, *Gapdh* (forward: AGCGAGACCCCACTAACATC and reverse: GGTTCACACCCATCACAAAC). Gene expression was assessed by SYBR green detection, normalized to expression of *Gapdh* and analyzed by the Comparative ΔΔC_T_ method to determine the difference in mutants relative to wild-type groups, as described [20].

### Statistical Analysis

Mean values and standard deviations (SD) or standard errors of mean (SEM) were calculated and analysis performed using unpaired Student’s *t*-test for independent samples in which the Bonferroni correction for multiple testing was applied [20]. A value of p<0.05 was considered significant for all analyses.

## Results

### Phenotypic Identification of Ectopic Calcification (EC) mice, Ecalc1 and Ecalc2

**Fig 1:**
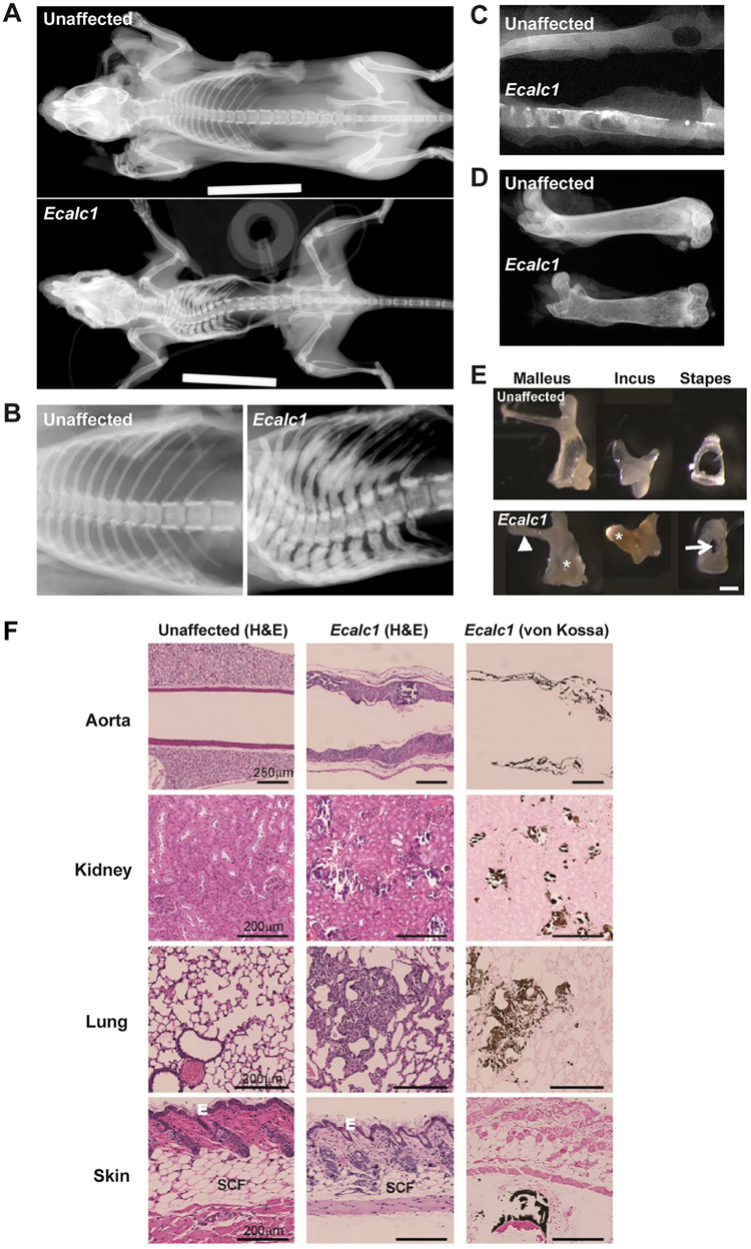
Phenotypic features of *Ecalc* mice. The *Ecalc1* and *Ecalc2* progeny had similar phenotypic features characterised by EC, growth retardation and reduced lifespan. In accordance with welfare guidelines, detailed phenotypic analysis was undertaken on the *Ecalc1* progeny only. Radiography of unaffected and affected *Ecalc1* mice (5 weeks old). (A) Affected *Ecalc1* mice have generalised reduction of soft tissue mass, kyphoscoliosis, widened zygomatic arches and irregular widened ribs. Dissected specimens revealing *Ecalc1* mice to have: (B) flattened vertebra and sclerotic ribs; (C) patchy opacifications within the aorta; (D) shortened radiolucent femora with cortical thinning: and (E) a shorter malleus with shortening and thickening of its manubrium (arrowhead), a reduction in size of the foramina between the crura (arrow) in the stapes, and the occurrence of ectopic calcification (asterisks) in the malleus and incus, when compared to unaffected littermates. Scale bar = 200μM. (F) Haematoxylin and eosin (H&E) and Von Kossa stained sections from affected *Ecalc1* mice and unaffected littermates. When compared to unaffected littermates, *Ecalc1* affected mice had: extensive aortic calcification leading to an irregular aortic lumen with widening of the medial and adventitial wall layers; necrotic regions within the renal parenchyma that were most pronounced around calcification foci; regions of dense calcification within the lungs that were associated with a reduced number of alveoli and inflammatory cell infiltrates; and skin calcification leading to reduced thickness of the epidermal (Epi) and subcutaneous fat (SCF) skin layers.

Phenotype analysis of two independent G3 progeny, derived from matings between parents and their offspring to yield autosomal recessive phenotypes, revealed, at 3 weeks of age, mice that were smaller, and with a hunched posture when compared to the unaffected littermates (*Ecalc1* = 28 affected (11 males and 17 females), 70 unaffected (41 males and 29 females); *Ecalc2* = 10 affected (5 males and 5 females), 37 unaffected (20 males and 17 females). Thus, ~29% of *Ecalc1* progeny and ~21% of *Ecalc2* progeny were affected with EC, consistent with the expected ~25% of affected progeny in an autosomal recessive trait. Further inheritance testing through breeding was not possible, because affected *Ecalc1* and *Ecalc2* mice had to be culled by 5 weeks of age, i.e. before puberty, due to poor health. All of the affected mice from *Ecalc1* and *Ecalc2* had a negative Preyer reflex, thereby indicating a hearing deficit. Radiography revealed that *Ecalc1* and *Ecalc2* mice had generalised reduction of soft tissue mass, thickened zygomatic arches, kyphoscoliosis, irregular widened ribs, shortened and radiolucent femora with cortical thinning, and opacifications affecting the aorta (Fig 1), consistent with EC. In accordance with welfare guidelines, further phenotypic analysis was undertaken on a single progeny only (*Ecalc1*), which was aged to a maximum 5 weeks. Histological analysis with von Kossa staining confirmed the presence of extensive aortic calcification leading to vessel occlusion; vascular and parenchymal renal calcification; pulmonary calcification with emphysematous changes; skin atrophy with reduced epidermal and subcutaneous fat thickness, and thickening and calcification of the auditory ossicles (Fig 1).

### Mapping of the *Ecalc1* and *Ecalc2* loci to chromosome 5G3 and identification of *Kl* coding-region mutations

**Fig 2:**
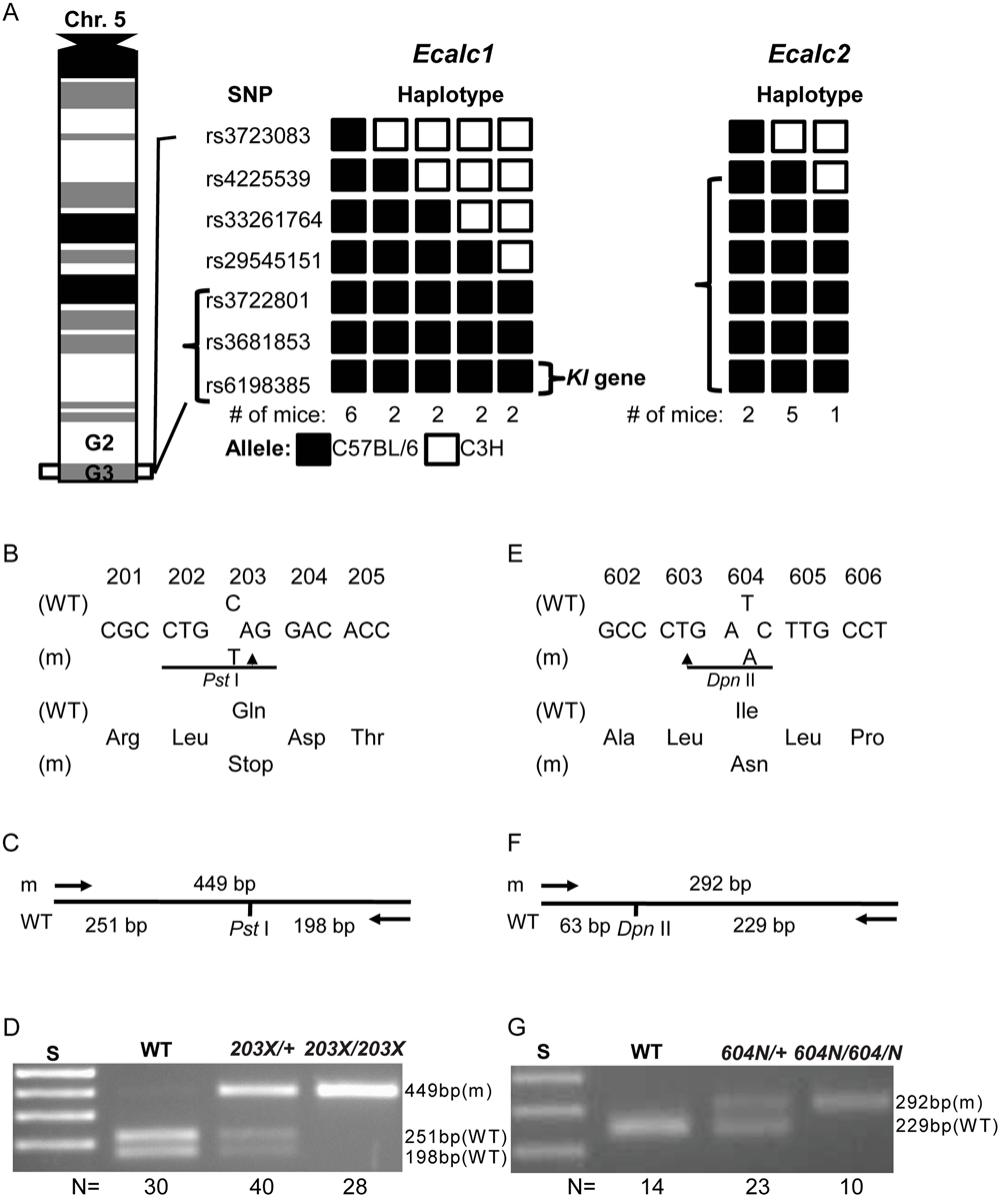
Mapping of *Ecalc1* and *Ecalc2* loci and identification of *Kl* mutations. (A) The *Ecalc1* and *Ecalc2* loci originated in a C57BL/6 ENU-mutagenised male and are hence inherited with C57BL/6 alleles. The *Ecalc1* locus was mapped to a 4.0 Mb region on chromosome 5 flanked by the rs29545151 and rs6198385 SNPs (square bracket). The *Ecalc2* locus was mapped to a 11.0 Mb overlapping interval between the rs4225539 and rs6198385 SNPs. The most likely candidate gene in these intervals was *Kl*, which is located between rs3681853 and rs6198385, and DNA sequence analysis of *Kl* was therefore undertaken. (B) A C to T transition at codon 203 was identified in affected *Ecalc1* mice. This altered the wild-type (WT) sequence (CAG), which encodes a glutamine (Gln) residue, to the mutant (m) sequence (TAG), which encodes a stop codon. The mutation resulted in the loss of a *PstI* restriction enzyme site (CTGCA/G), and this was used to confirm the mutation. (C) A 449bp PCR-amplified product cleaved with *PstI* is predicted to yield 251bp and 198bp products from the WT allele, whereas the mutant allele remains undigested with a resultant 449bp product. (D) Gel electrophoresis of PCR products digested with *PstI* showing DNA from WT to be homozygous for the 251bp and 198bp bands; *kl* ^*203X/+*^ unaffected mice to be heterozygous for the 449bp and 251bp + 198bp bands; and *kl* ^*203X/203X*^ affected mice to be homozygous for the mutant 449bp band. (E) A T to A transversion was identified at codon 604 in affected *Ecalc2* mice. This altered the WT sequence (ATC), which encodes an isoleucine (Ile) residue, to the m sequence (AAC), which encodes an asparagine (Asn) residue. The mutation resulted in the loss of a *DpnII* site (/GATC), and this was used to confirm the mutation. (F) A 292bp PCR-amplified product cleaved with *DpnII* is predicted to yield 229bp and 63bp products from the WT allele, whereas the mutant allele remains undigested with a resultant 292bp product. (G) Gel electrophoresis of PCR products digested with *DpnII* showing DNA from WT to be homozygous for the 229bp and 63bp bands; *kl* ^*604N/+*^ unaffected mice to be heterozygous for the 292bp and 229bp + 63bp bands; and *kl* ^*604N/604N*^ affected mice to be homozygous for the mutant 292bp band. S is the size marker (1Kb ladder).

**Fig 3:**
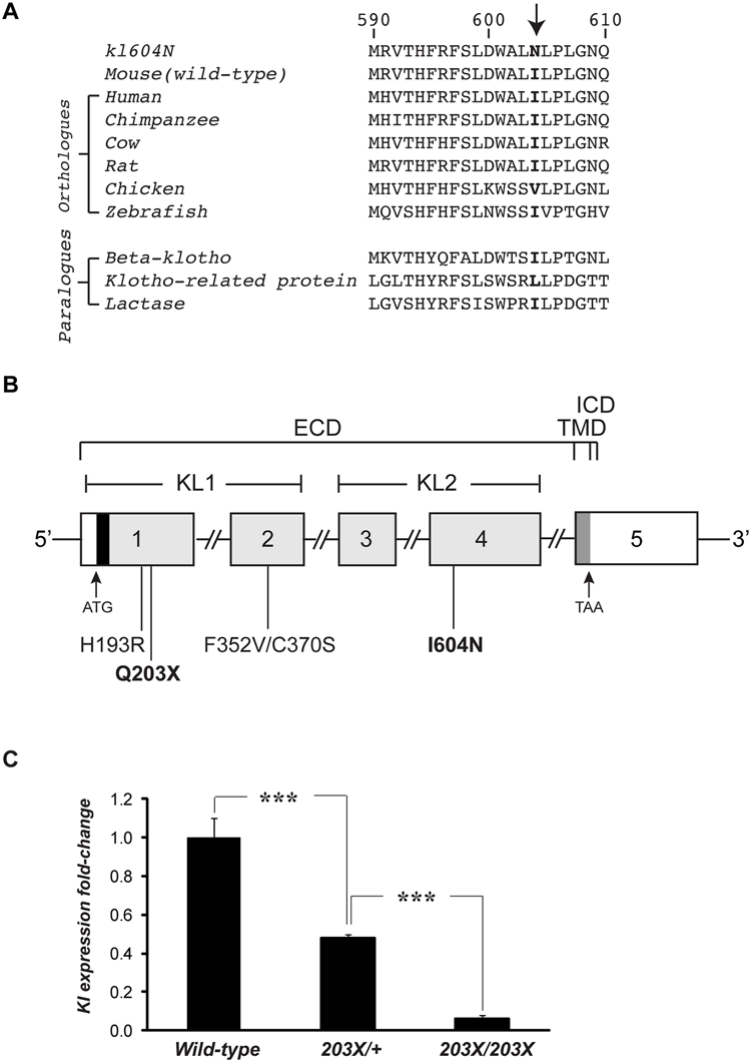
Protein sequence alignment, mutation location and renal *Kl* gene expression. (A) Protein sequence alignment (NCBI BLAST) of klotho residues in orthologues from 7 species and 3 paralogues, revealed evolutionary conservation of the Ile604 residue that is mutated to Asn604 in *kl* ^*604N*^ mice. The Ile604 residue (arrow) is either conserved as an Ile residue amongst orthologues and paralogues or conservatively substituted with a Leu or Val residue. (B) Schematic representation of the genomic organisation of the human and mouse klotho gene showing location of reported pathogenic coding-region mutations causing TC in man, and common variants associated with a human ageing phenotype [9, 29]. The klotho gene consists of 5 exons (1–5). The start (ATG) and stop (TAA) codons are located in exons 1 and 5, respectively. The 5’ portion of exon 1, and the 3’ portion of exon 5 are untranslated (open boxes). The klotho protein N-terminal signal peptide is encoded by the 5’ portion of exon 1 (black). Exons 1–4 encode the extracellular KL1 and KL2 repeat regions (light grey) that have homology to β-glycosidases. The 5’ portion of exon 5 (dark grey) encodes the single-pass transmembrane domain and short intracellular domain. The sites of coding-region klotho mutations and variants are indicated. The previously reported loss-of-function His193Arg (H193R) mutation and common Phe352Val/Cys370Ser (F352V/C370S) double variant that leads to a gain-of-function are located in the extracellular KL1 domain. Klotho mutations identified in the *kl* ^*203X*^ *and kl* ^*604N*^ mice are highlighted in bold. The *kl* ^*203X*^ Gln203Stop (Q203X) mutation is located in the KL1 domain and predicted to lead to a truncated protein lacking the intracellular, transmembrane and extracellular KL2 domains. The mutant Gln203Stop klotho transcript is likely to undergo nonsense-mediated decay. The *kl* ^*604N*^ Ile604Asn (I604N) mutation represents the first pathogenic variant identified in the KL2 domain. ECD, extracellular domain; TMD, transmembrane domain; ICD, intracellular domain. (C) Analysis of renal *Kl* gene expression. RNA from whole kidneys was extracted from wild-type (WT) littermates (N = 3 males), *kl* ^*203X/+*^ (N = 3 males) and *kl* ^*203X/203X*^ (N = 3 males) mice, aged 4–5 weeks. Quantitative reverse transcriptase-PCR (qRT-PCR) was used to study the expression of *Kl*. Samples were analysed in triplicate, and mRNA levels were normalized to *Gapdh* and expressed as fold-change (mean ± SEM) compared to wild-types. The expression of *Kl* was significantly reduced in *kl* ^*203X/+*^ and *kl* ^*203X/203X*^ mice when compared to wild-type littermates. ***p<0.001.

Genome-wide analysis using 91 SNP sets and DNA samples from 12 affected *Ecalc1* (9 males and 3 females) mice localised the *Ecalc1* locus to a 4.0 Mb region (between 147 and 151 Mb) flanked by rs29545151 and rs6198385 on chromosome 5G3 that contained 68 genes, of which *klotho* (*Kl*) was the most likely candidate (Fig 2). Similar genome-wide mapping using DNA samples from 8 affected *Ecalc2* (5 males and 3 females) mice localised the *Ecalc2* locus to a 11.0 Mb region (between 140 and 151 Mb) flanked by rs4225539 and rs6198385 on chromosome 5G3, that contained 198 genes, and amongst this *Kl* was the most likely candidate. DNA sequence analysis of the entire *Kl* 3045-bp coding-region and exon-intron boundaries was therefore undertaken in affected *Ecalc1* and *Ecalc2* mice and this revealed *Ecalc1* mice to harbour a C to T transition at codon 203 within exon 1, which altered a glutamine (Gln) amino acid residue to a stop codon; and *Ecalc2* mice to harbour a T to A transversion at codon 604 in exon 4, which altered an isoleucine (Ile) residue to an asparagine (Asn) residue (Figs 2 and 3). No other DNA sequence abnormalities of the *Kl* gene were identified. The Gln203Stop nonsense mutation resulted in the loss of a *PstI* restriction enzyme site, whilst the Ile604Asn missense mutation resulted in a loss of a *DpnII* restriction enzyme site, and these were used to confirm the presence of the mutations (Fig 2). The Ile604Asn *Kl* missense substitution affects an evolutionary conserved residue and represents the first mutation to be identified in the extracellular KL2 domain (Fig 3).

### Functional characterization of the Gln203Stop and Ile604Asn *Kl* mutations in *Ecalc1* and *Ecalc2* mice

**Fig 4:**
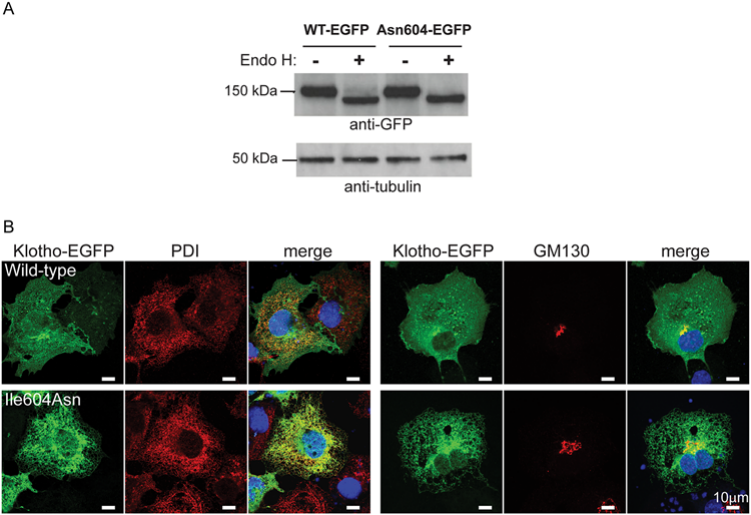
Retention of *kl* ^*604N*^-mutant Asn604 klotho protein within the endoplasmic reticulum. (A) Western blot analysis of cell lysates from COS-7 cells transfected with the wild-type (WT) or *kl* ^*604N*^-mutant (Asn604) EGFP-tagged constructs. Wild-type and mutant klotho proteins were detected in equal amounts in the cell lysates using the anti-EGFP antibody. Treatment with Endo H revealed undigested wild-type klotho proteins whereas all the mutant Asn604 klotho protein was Endo H-sensitive, suggesting that the mutant Klotho protein is retained in the endoplasmic reticulum. Anti-tubulin antibody was used as a loading control. (B) COS-7 cells were transiently transfected with either wild-type-EGFP or mutant-EGFP (Asn604) constructs, and counterstained with anti-PDI antibody, which immunostains the ER (red), or anti-GM130 antibody, which immunostains the Golgi apparatus (red). DAPI was used to stain the nucleus (blue). Wild-type klotho is expressed at the plasma membrane, with some co-localizing with PDI and GM130, whereas the mutant klotho co-localizes with PDI and is thus predominantly retained within the ER.

The Gln203Stop mutation of the *Ecalc1* mice (*kl* ^*203X*^ *)* was predicted to result in a truncated klotho protein that would be non-functional as it lacked a transmembrane domain and most (>75%) of the extracellular domain, and would be thus unable to localize to the plasma membrane and act as a co-receptor for FGF23. Moreover, it would seem likely that the Gln203Stop mutant transcript would undergo nonsense-mediated decay and the expression of *Kl* transcripts in wild-type and *kl* ^*203X*^ mice was therefore investigated using quantitative reverse transcriptase-PCR (qRT-PCR) of RNA obtained from the kidneys of wild-type and *kl* ^*203X*^ mice. This revealed homozygous-affected (*kl* ^*203X/203X*^) mice to have a 17-fold reduction of *klotho* expression when compared to wild-type mice (p<0.001), whilst heterozygous (*kl* ^*203X/+*^) mice, which did not harbour the affected phenotype, had a 2-fold reduction of *klotho* expression compared to wild-types (p<0.001) (Fig 3C), consistent with the occurrence of nonsense-mediated decay of mutant *Kl* transcripts in *kl* ^*203X*^ mice. The effects of the missense Ile604Asn *Kl* mutation, which represents the first identified mutation involving the KL2 domain (Fig 3) are difficult to predict. The only other previously described klotho missense mutation, (His193Arg), to date, is located in the KL1 domain and this has been demonstrated to impair klotho protein stability [9]. We therefore investigated whether the Ile604Asn *Kl* mutation may impair the expression and cellular processing of klotho by *in vitro* transient transfection of wild-type or *Ecalc2 (kl* ^*604N*^ *)*-mutant enhanced green fluorescent protein (EGFP)-tagged *Kl* cDNA constructs in COS-7 cells (Fig 4). Western blot analysis of whole cell lysates obtained from transiently transfected COS-7 cells demonstrated similar levels of expression of wild-type or *kl* ^*604N*^-mutant EGFP-tagged *Kl* constructs (Fig 4A). Treatment of transfected cell lysates with the deglycosylating enzyme, Endo H, which cleaves high mannose N-linked glycans that are introduced in the endoplasmic reticulum (ER), revealed the presence of Endo H-sensitive products in both cell lysates (Fig 4A), thereby indicating that wild-type and *kl* ^*604N*^-mutant Asn604 klotho proteins had high mannose structures, and thus entered the ER lumen. However, lysates from cells expressing the *kl* ^*604N*^-mutant Asn604 klotho protein lacked Endo H-resistant products, which were present in cells expressing wild-type klotho (Fig 4A). This suggests that the *kl* ^*604N*^-mutant Asn604 klotho protein does not undergo the complex oligosaccharide modifications that occur in the Golgi apparatus, but may instead be retained in the ER. Immunofluorescence and confocal microscopy was undertaken to determine the sub-cellular localization of wild-type and mutant klotho proteins. Wild-type (Ile604) klotho-EGFP was found to be expressed at the plasma membrane, with some intracellular expression co-localizing with the ER marker, protein disulphide isomerase (PDI) and the Golgi matrix protein (GM130), whereas the *kl* ^*604N*^-mutant Asn604 klotho protein predominantly co-localized with the ER marker, PDI (Fig 4B), thereby indicating impaired trafficking and ER retention of the mutant protein. Thus, the Gln203Stop and Ile604Asn *Kl* variants were demonstrated to be pathogenic mutations that impaired the expression or subcellular localization of the klotho protein, and provide an explanation for the severe phenotypes and lack of viability beyond 5 weeks for both the *kl* ^*203X/203X*^ and *kl* ^*604N/604N*^ mice.

### Bone and Mineral Metabolic phenotype of *kl^203X^* mice

**Fig 5:**
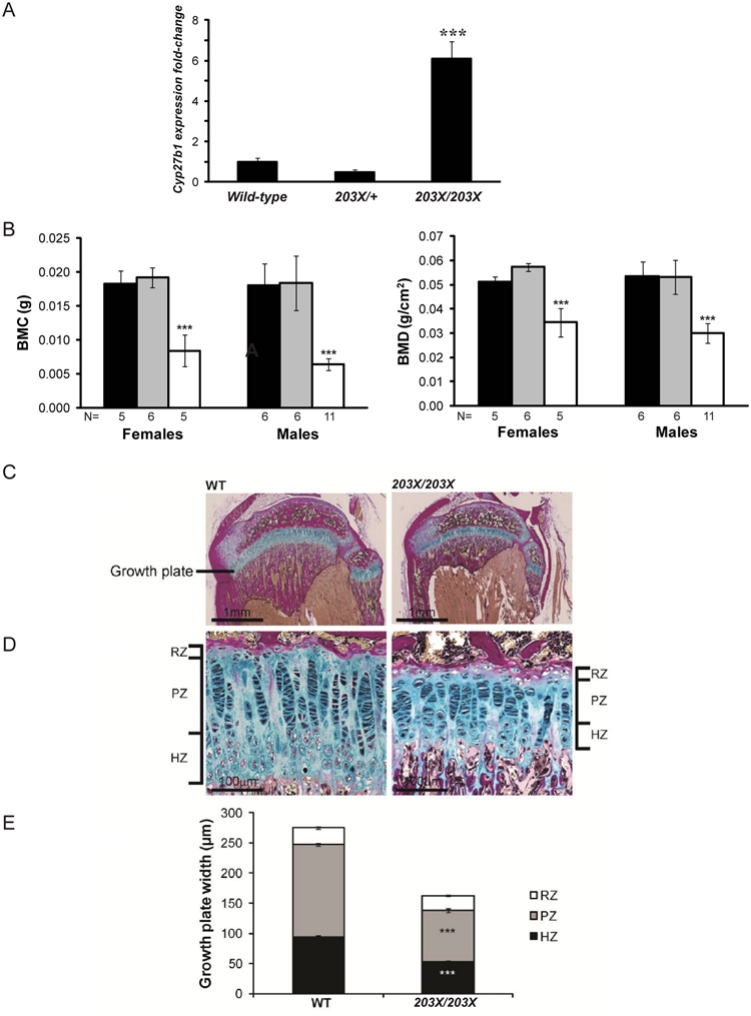
Bone and mineral metabolic phenotype of *kl* ^*203X*^ mice. (A) Analysis of renal *Cyp27b1* gene expression. RNA from whole kidneys was extracted from wild-type (WT) littermates (N = 3 males), *kl* ^*203X/+*^ (N = 3 males) and *kl* ^*203X/203X*^ (N = 3 males) mice, aged 4–5 weeks. Quantitative reverse transcriptase-PCR (qRT-PCR) was used to study the expression of *Cyp27b1*. Samples were analysed in triplicate, and mRNA levels were normalized to *Gapdh* and expressed as fold-change (mean ± SEM) compared to wild-types. The expression of *Cyp27b1* was significantly increased in *kl* ^*203X/203X*^ mice, but not *kl* ^*203X/+*^ mice, when compared to wild-type littermates. ***p<0.001. (B) DXA analysis of dissected femora from 5 week old mice revealed significantly reduced bone mineral content (BMC) and bone mineral density (BMD) in *kl* ^*203X/203X*^ mice when compared to *kl* ^*203X/+*^ and wild-type littermates. ***p<0.001. (C-E) Histological analysis of wild-type and *kl* ^*203X/203X*^ growth plates were undertaken by staining paraffin embedded proximal tibial sections with Alcian blue (cartilage) and van Gieson (osteoid). This revealed growth plate narrowing in *kl* ^*203X/203X*^ mice (C), which is due to a significantly reduced height of the proliferating zone (PZ) and hypertrophic zone (HZ) (D and E). The height of the reserve zone (RZ) was unaffected. ***p<0.001.

*Kl* gene abnormalities in human and mice are associated with dysregulation of bone and mineral metabolism, as well as EC (Table 1). The mineral metabolic abnormalities include hypercalcaemia, hyperphosphataemia, increased circulating concentrations of 1,25 dihydroxyvitamin D and FGF23 as well as osteopaenia. The bone and mineral metabolic abnormalities resulting from the ENU-induced *Kl* mutations were therefore investigated in one of the models, *kl* ^*203X*^, which was due to a nonsense mutation, Gln203Stop, and hence likely to be more severe than the missense mutation, Ile604Asn of *kl* ^*604N*^ mice, although both *kl* ^*203X*^ and *kl* ^*604N*^ mutant mice were not viable beyond 5 weeks of age. Analysis of plasma from 4–5 week old mice did not reveal any significant differences between heterozygous affected (*kl* ^*203X/+*^) mice and wild-type littermates (Table 2). However, male and female homozygous affected (*kl* ^*203X/203X*^) mice had significantly increased plasma concentrations of phosphate and alkaline phosphatase activity, but were normocalcaemic (Table 2). There were also gender-specific differences, as female *kl* ^*203X/203X*^ mice had significantly lower plasma glucose concentrations compared to female wild-types, whereas male *kl* ^*203X/203X*^ mice had significantly reduced plasma albumin concentrations when compared to male wild-types (Table 2). As male and female *kl* ^*203X/203X*^ mice were revealed to be hyperphosphataemic, measurements of plasma 1,25 dihydroxyvitamin D and intact FGF23 were undertaken. The plasma concentrations of 1,25 dihydroxyvitamin D were significantly elevated in *kl* ^*203X/203X*^ mice compared to wild-types (Table 2), consistent with a significantly increased (6-fold) expression of vitamin D-1α-hydroxylase (*Cyp27b1*) in *kl* ^*203X/203X*^ mice when compared to wild-type littermates (p<0.001) (Fig 5A). Plasma intact FGF23 concentrations were markedly raised in male and female *kl* ^*203X/203X*^ mice, such that the values obtained were above the upper limit of assay detection (Table 2). Radiography showed the femora of *kl* ^*203X/203X*^ mice to have reduced radiodensity and cortical thickness, consistent with osteopaenia (Fig 1D), and DXA analysis of femora harvested from mice aged 5 weeks demonstrated male and female *kl* ^*203X/203X*^ mice, but not *kl* ^*203X/+*^ mice, to have significant reductions in bone mineral content (BMC) (p<0.001) and bone mineral density (BMD) (p<0.001), when compared to wild-type littermates (Fig 5B). However, the size of the femora from *kl* ^*203X/203X*^ mice was reduced when compared to *kl* ^*203X/+*^ and wild-type mice, and this may contribute to the observed reductions in BMC and BMD, as measured by DXA [30]. The basis of the shortened long bones of *kl* ^*203X/203X*^ mice (Fig 1D) was investigated by examining endochondral ossification in the proximal tibiae from 5-week old mice. This revealed narrowing of the growth plate region (Fig 5C) secondary to an ~45% reduction in the width of both the proliferative and hypertrophic epiphyseal zones of affected mice (Fig 5D and 5E).

**Table 2:** Plasma analysis of ~5 week old *kl* ^*203X*^ mice.

	Females			Males		
	Wild-type	*kl* ^*203X/+*^	*kl* ^*203X/203X*^	Wild-type	*kl* ^*203X/+*^	*kl* ^*203X/203X*^
**Calcium** ^**a**^ **(mmol/L)**	2.39±0.09 (n = 13)	2.36±0.04 (n = 15)	2.27±0.10 (n = 11)	2.24±0.07 (n = 14)	2.40±0.06 (n = 19)	2.34±0.09 (n = 16)
**Phosphate (mmol/L)**	3.37±0.18 (n = 14)	3.52±0.11 (n = 15)	4.72±0.14*** (n = 9)	3.47±0.2 (n = 14)	3.66±0.17 (n = 19)	4.20±0.16** (n = 16)
**ALP (U/L)**	194±19 (n = 14)	225±8 (n = 15)	501±78.7*** (n = 11)	224±31 (n = 14)	185±13 (n = 19)	432±40** (n = 16)
**Albumin (g/L)**	22.6±2.0 (n = 13)	25.5±0.5 (n = 15)	23.6±1.0 (n = 11)	24.3±0.5 (n = 14)	24.2±0.7 (n = 19)	21.8±0.6* (n = 16)
**ALT (U/L)**	27.6±4.7 (n = 12)	46.4±13.7 (n = 15)	45.6±7.6 (n = 8)	40.89±9.50 (n = 12)	35.66±6.70 (n = 16)	17.99±2.00 (n = 4)
**Urea (mmol/L)**	8.19±0.52 (n = 9)	7.66±0.54 (n = 7)	11.76±2.31 (n = 4)	7.15±0.58 (n = 7)	8.22±0.57 (n = 9)	9.44±0.52 (n = 6)
**Glucose (mmol/L)**	11.9±0.8 (n = 12)	11.5±0.5 (n = 15)	5.5±0.5*** (n = 10)	10.0±0.6 (n = 13)	11.6±1.0 (n = 16)	7.2±1.4 (n = 12)
**1,25(OH)**_**2**_ **D (pmol/L)**	96±20 (n = 6)	100±13 (n = 6)	309±70** (n = 4)	115±16 (n = 5)	156±53 (n = 5)	471^b^, 375^b^ (n = 2)
**FGF23 (pg/mL)**	256±35 (n = 14)	363±43 (n = 13)	↑^c^ (n = 9)	436±90 (n = 8)	410±39 (n = 16)	↑^c^ (n = 14)

Values are expressed as mean±SEM.

^a^Plasma calcium concentrations were normalized to the mean plasma albumin concentration.

^b^Plasma 1,25-dihydroxyvitamin D concentrations were measured in two male *kl* ^*203X/203X*^ mice, and the value for each individual mouse, which is provided in the table, is >5 SD above the mean of the male wild-type mice.

^c^Male and female *kl* ^*203X/203X*^ mice had plasma FGF23 concentrations that were above the upper limit of assay detection, indicative of marked FGF23 elevations. ↑, increased; ALP, alkaline phosphatase activity; ALT, alanine aminotransferase; FGF23, fibroblast growth factor; 1,25(OH)_2_D, 1,25-dihydroxyvitamin D.

*p<0.05.

**p<0.01,

***p<0.001.

## Discussion

Our study describes two EC mouse mutants, designated *kl* ^*203X*^ and *kl* ^*604N*^, which harbour germline coding-region nonsense and missense *Kl* mutations, respectively, that were induced by ENU. This chemical mutagen is known to induce multiple mutations simultaneously [18]. However, it was unlikely that another genetic defect within the 11.0 Mb region that was established as the location of the *Ecalc1* and *Ecalc2* loci (Fig 2A) could be the underlying cause of EC. Indeed, the likelihood of another genetic cause was estimated to be <0.01, based on the following reasoning. The nominal ENU induced base pair mutation rate for potentially functional mutations has been estimated to be 1 in 1.82 Mb of coding DNA in the F1 founder animals [31], and given that <2.5% of the mouse genome is coding, it has been calculated that the probability of two functional mutations arising within a 5.0 Mb genomic region is <0.002 [32]; thus the likelihood of functional mutations arising in addition to the *Kl* mutations within the 11.0 Mb containing the *Ecalc1* and *Ecalc2* loci is <0.005. This indicates that the *Ecalc1* Gln203Stop and *Ecalc2* Ile604Asn *Kl* mutations, which were shown to result in nonsense-mediated *Kl* transcript decay and ER retention of the mutant klotho protein, respectively (Figs 3C and 4), are highly likely to be the sole genetic defects causing EC in these mouse models. Indeed, an analysis of the 198 genes within the 11.0 Mb region that contains the *Ecalc1* and *Ecalc2* loci (Fig 2A) did not reveal any known phosphate-regulating genes (S1 Dataset).

*ENU-derived kl* ^*203X/203X*^ mice were confirmed as a model for klotho deficiency as they had markedly reduced renal *Kl* expression in association with hyperphosphataemia, raised circulating 1,25 dihydroxyvitamin D and FGF23 concentrations, and widespread EC. The heterozygous affected (*kl* ^*203X/+*^) mice did not exhibit any clinical or biochemical abnormalities, and these findings are in keeping with other TC mouse models, in which heterozygous mice are unaffected [10–12, 22], and consistent with TC being an autosomal recessive disorder [6]. The phenotypes of *kl* ^*203X/203X*^ mice are in keeping with reported klotho mouse mutants and a TC patient harbouring a germline missense *KL* mutation (Table 1) [9, 11, 22, 33, 34], and highlight the central importance of klotho in systemic mineral metabolism. The *kl* ^*203X/203X*^ mice also share a complex skeletal phenotype with the klotho mouse models and TC patient that is characterised by the combined occurrence of osteopaenia and sclerotic or hyperostotic lesions [9, 11, 22]. Bone histology of klotho deficient mice previously revealed the osteopaenia to be caused by cortical thinning of long bone diaphyses, whereas the epiphyses may paradoxically have an increased BMD and trabecular bone volume [35, 36]. Moreover, hypomineralisation and increased osteoid formation has been previously observed within the metaphyseal regions of klotho deficient mice [22, 37], and although we did not assess osteoid formation by tetracycline labelling due to the young age and poor health of affected mice, our findings of elevated alkaline phosphatase activity in *kl* ^*203X/203X*^ mice are consistent with klotho deficiency being associated with an osteomalacic phenotype. Furthermore, the shortened femora of *kl* ^*203X/203X*^ mice was revealed to be associated with narrowing of the growth plate hypertrophic and proliferative zones, and this may be a consequence of hyperphosphataemia-induced chondrocyte apoptosis [38]. The present study has also revealed the hearing deficit of *kl* ^*203X/203X*^ mice to represent a novel skeletal manifestation of klotho deficiency that is characterised by sclerosis of the auditory ossicles. Similar abnormalities of the auditory ossicles have recently been reported in *Fgf23* null mice [39]. However, hearing deficits have not been described in TC patients harbouring *FGF23* or *GALNT3* mutations, or in the sole TC patient reported to date with a *KL* mutation [8, 9, 40]. Moreover, periarticular calcification, which is a hallmark feature of TC in humans, was not detected in *kl* ^*203X/203X*^ mice or reported in other TC mouse models [10–12, 22]. Periarticular calcification has been described in EC mouse models that harbour abnormalities of the *Enpp1* or *Ank* genes [41, 42]. However, these mice were assessed in adulthood (≥4 months of age), whilst TC mice with ablated *Fgf23* or *Kl* alleles do not survive past 3 months of age [11, 12, 22]. Thus, the lack of periarticular calcification in TC mice may potentially be a consequence of their young age or represent a species-specific difference. The *kl* ^*203X/203X*^ mice displayed phenotypic differences to the reported mouse models and TC patient with klotho mutations (Table 2) [9, 11, 22, 33, 34]. In particular, mild hypercalcaemia, which had been reported to arise in association with increased renal synthesis of 1,25 dihydroxyvitamin D in klotho deficient mice [43], or from hyperparathyroidism as occurred in the *KL* mutation patient [9], was absent in *kl* ^*203X/203X*^ mice. We postulate that *kl* ^*203X/203X*^ mice compensate for the hypercalcaemic effects of increased circulating 1,25 dihydroxyvitamin D concentrations by mounting a hypercalciuric response. Although urinary calcium excretion could not be assessed because of the poor health and reduced life of the *kl* ^*203X/203X*^ *mice*, renal parenchymal calcification was detected (Fig 1F), which may be suggestive of hypercalciuria. Moreover, male *kl* ^*203X/203X*^ mice were hypoalbuminaemic, and this finding, which may represent a malnourished state, has not been reported in other klotho deficient mice or in the TC patient with a *KL* mutation [9, 11, 22]. The ENU-derived *kl* ^*203X/203X*^ *mice* and other klotho deficient mouse models have a more severe phenotype than that reported for the patient harbouring a *KL* mutation [9]. Thus, klotho deficient mice have a reduced lifespan and develop multi-systemic abnormalities that include: growth retardation, hypoglycaemia due to increased insulin sensitivity [11, 44], a generalised reduction of white adipose tissue that may be a consequence of impaired adipocyte maturation [11, 45], and senescence-related traits such as pulmonary emphysema and skin atrophy, which are considered to arise from calcium deposition that induces loss of collagen fibres and progressive deterioration of tissue architecture [11, 22, 46]. These extra-skeletal manifestations appear to be species-specific, as they have not been reported in patients with TC [8, 9, 40], and intriguingly it has been postulated that humans may be able to partially compensate for the effects of klotho deficiency on phosphate homeostasis, thereby minimizing the severity of the multi-systemic hyperphosphataemic phenotype [9]. Indeed, hyperphosphataemia appears to be central to the pathogenesis of many of these klotho mouse mutant traits, as they are successfully rectified following administration of a reduced phosphate diet [47], or by genetic intervention such as ablation of *Cyp27b1* to lower circulating phosphate concentrations [34]. The precise cause of premature death in the ENU-derived klotho mutants and in other klotho deficient mice has not been elucidated. Possible aetiologies include extensive vascular calcification leading to arterial occlusion or arteriosclerosis-mediated hypertension and cardiac failure, hypoglycaemia, or respiratory insufficiency due to emphysema [11, 22].

The ENU derived *kl* ^*203X*^ mice harbour a germline Gln203Stop *Kl* mutation that was predicted to lead to nonsense-mediated decay of the *Kl* transcript or result in a non-functional truncated protein lacking the transmembrane and extracellular KL2 domains. The functional consequences of the *kl* ^*604N*^ Ile604Asn *Kl* mutation, which is located in the extracellular KL2 domain, were more difficult to predict, as the molecular basis of klotho protein activity has yet to be elucidated and crystal structure information is not available. Some insights into klotho structure-function have been provided by studies of missense substitutions located in the extracellular KL1 domain (Fig 3B). Indeed, *in vitro* functional expression analyses of the human TC-associated His193Arg klotho mutation and a common polymorphic klotho variant comprised of a double missense substitution (Phe352Val/Cys370Ser), which is associated with an ageing phenotype [29], have revealed key KL1 residues that participate in protein-protein interactions with the FGFR and FGF23; thus the His193Arg mutation led to a decrease in the affinity of klotho for FGFR and FGF23 [9], whereas the common Phe352Val/Cys370Ser variant enhanced this interaction and promoted FGF23-mediated signal transduction [48]. The extracellular KL1 and KL2 domains may also facilitate the cleavage of oligo- and polysaccharides as they each share up to 40% homology with β-glycosidase enzymes, and the klotho protein has previously been revealed to activate the renal TRPV5 calcium channel by removing sialic acid residues from its N-linked glycan chains [49]. The reported His193Arg mutation is located within the predicted catalytic pocket of the KL1 domain, and is considered to destabilise the tertiary structure of this domain, thereby leading to reduced klotho expression and secretion [9]. Our *in vitro* functional expression studies demonstrated the Ile604Asn klotho mutant to be retained within the ER, highlighting that this missense mutation also likely impairs protein folding and stability. Analysis of the crystal structure of a human paralogue known as klotho-related protein, which has β-glucosidase activity [50], revealed the wild-type Ile604 residue to be located within a region of the KL2 domain that may participate in glucosidase activity by promoting transition states involved in the hydrolysis of β-glucosides [51], and we postulate that the substitution of the non-polar Ile residue with a polar Asn residue may alter the structural conformation and stability of the KL2 domain.

In summary, our study has established two novel mouse models with EC and phenotypic features associated with premature ageing, which are due to ENU induced nonsense (Gln203Stop) and missense (Ile604Asn) mutations of the *Kl* gene. These mouse models will help to further elucidate the molecular basis of klotho function and characterise the role of klotho in the renal regulation of phosphate metabolism.

## Supporting Information

S1 Dataset List of genes within the *Ecalc1* and *Ecalc2* candidate region. (XLS)
